# Celiac Disease Causes Epithelial Disruption and Regulatory T Cell Recruitment in the Oral Mucosa

**DOI:** 10.3389/fimmu.2021.623805

**Published:** 2021-02-25

**Authors:** Javier Sanchez-Solares, Luis Sanchez, Carmela Pablo-Torres, Celso Diaz-Fernandez, Poul Sørensen, Domingo Barber, Cristina Gomez-Casado

**Affiliations:** ^1^ Institute of Applied Molecular Medicine, Hospitals Madrid (HM) Group, San Pablo-CEU University, Madrid, Spain; ^2^ Service of Gastroenterology, University Hospital San Agustin (HUSA), Aviles, Spain; ^3^ Department of Otolaryngology Head and Neck Surgery, University Hospital San Agustin (HUSA), Aviels, Spain; ^4^ Allero Therapeutics BV, Rotterdam, Netherlands; ^5^ Department of Biomedicine, Aarhus University, Aarhus, Denmark; ^6^ ARADyAL-RD16/0006/0015, Thematic Network and Cooperative Research Centers, ISCIII, Madrid, Spain

**Keywords:** celiac disease, oral mucosa, remodeling, regulatory T cells, immunotherapy, tolerance induction, immune mediated disorders, autoimmune disease

## Abstract

Celiac disease (CD) is a chronic autoimmune disease characterized by an immune-triggered enteropathy upon gluten intake. The only current treatment available is lifelong Gluten Free Diet (GFD). Several extraintestinal manifestations have been described in CD, some affecting the oral mucosa. Thus, we hypothesized that oral mucosa could potentially be a target for novel biomarkers and an administration route for CD treatment. Six *de novo* diagnosed and seven CD patients under GFD for at least 1 year were recruited. Non-celiac subjects (n = 8) were recruited as control group. Two biopsies of the cheek lining were taken from each subject for mRNA analysis and immunohistochemical characterization. We observed a significant decrease in the expression of epithelial junction proteins in all CD patients, indicating that oral mucosa barrier integrity is compromised. FoxP3+ population was greatly increased in CD patients, suggesting that Tregs are recruited to the damaged mucosa, even after avoidance of gluten. Amphiregulin mRNA levels from Peripheral Blood Mononuclear Cells (PBMCs) and epithelial damage in the oral mucosa correlated with Treg infiltration in all the experimental groups, suggesting that recruited Tregs might display a “repair” phenotype. Based on these results, we propose that oral mucosa is altered in CD and, as such, might have diagnostic potential. Furthermore, due to its tolerogenic nature, it could be an important target for oral immunotherapy.

## Introduction

Celiac disease (CD) is characterized by an immune-mediated chronic enteropathy of the small intestine, triggered by the ingestion of gluten in genetically predisposed individuals. The prevalence of CD is 1–3% worldwide. Although this prevalence varies from country to country, i.e. 1% in Western countries, due to environmental, autoimmune, and genetic factors. CD is regarded as one of the most common genetic disorders, as virtually all the patients are HLA-DQ2+ and/or HLA-DQ8+ (1–4).

It is characterized by the atrophy of the villi of the small intestinal mucosa, which entails a syndrome of nutrient malabsorption (1). CD diagnosis is currently based on several features that include, besides the HLA haplotypes, serological markers (IgA anti-endomysial and/or IgA anti-tissue transglutaminase -tTG2-) and gluten-induced intestinal morphological changes. These changes have led to the Marsh classification score based on a) lymphocyte infiltrates at the intraepithelial compartment; b) crypt hyperplasia; and c) villous atrophy (5). Therefore, CD diagnosis requires a biopsy from the small intestine, a sampling technique that is invasive and entails possible complications for the patients.

The typical form of the disease is a malabsorption syndrome with chronic diarrhea, abdominal pain, distention, and weight loss (6). However, due to the deficiencies of the nutrients absorbed in the small intestine such as iron, folic acid, or vitamin B12, atypical forms include extra-intestinal manifestations as iron-deficiency anemia, dermatitis herpetiformis, osteoporosis, and osteopenia. Lesions in the oral and gingival mucosa, tongue, palate, tooth, and enamel are also frequently associated to CD. In fact, recurrent aphthous stomatitis and atrophic glossitis are present in more than 20% of CD patients (6–8).

Currently, the only available treatment for CD is the adherence to a lifelong GFD. After gluten removal, an improvement in clinical symptoms and intestinal histological findings is observed. However, some patients find this diet cumbersome, as it is more expensive and socially restrictive than ordinary diets (9, 10). In this regard, several adverse effects and negative psychosocial implications have been reported (11). As a result, an increasing number of trials have recently begun to explore alternative therapeutic strategies, such as enzymes designed to digest gluten, the use of inhibitors of paracellular permeability to decrease the migration of gluten peptides into the lamina propria, binding of gluten by polymers, the use of tissue transglutaminase (tTG2) inhibitors, or the modulation of cytokine production. Interestingly, gluten tolerization by antigen specific immunotherapy is also being recently pursued (12). The latter approach is based on allergy immunotherapy (AIT), which is a well described tolerance‐inducing and disease modifying treatment for allergic diseases that acts through several mechanisms, including the generation of B and T regulatory responses (13, 14). Among AIT strategies, sublingual immunotherapy (SLIT) is both a safe and effective treatment for allergic rhinitis and asthma (15–17). Recent accumulating evidence suggests that a possible “mouth-gut axis” may exist in the pathogenesis of gastrointestinal diseases (18), and as such, oral mucosa may be a key target organ for the development of CD-specific immunotherapy.

The oral mucosa lines the inside of the mouth and consists of primarily non-keratinized stratified squamous epithelia, and an underlying connective tissue, the lamina propria, which is highly vascularized (19). Epithelial integrity is maintained by junction protein complexes, such as adherens junctions (AJs) and tight junctions (TJs) (20). Besides being a physical barrier, the oral mucosa contains immune cells that maintain mucosal homeostasis. In fact, the mucosal epithelium plays a key role in the immune regulatory system of the oral mucosa, whose function is primarily tolerogenic (19, 21, 22). When the epithelium is disrupted, epithelium-derived cytokines, such as thymic stromal lymphopoietin (TSLP), IL-25, and IL-33, are released. Another cytokine recently associated with epithelial tissue damage is periostin, which provides signals for tissue development and remodeling (23–25). Factors such as platelet-activating factor (PAF) can contribute to barrier remodeling by activating epithelial cells to release IL-33 (26). IL-33, in turn, signals for epithelial remodeling associated inflammation and the recruitment of Tregs (27). The recruited Tregs contribute to mucosal homeostasis by promoting wound healing and repair processes. Recruited “repair” Tregs express and/or produce repair factors such as amphiregulin and keratinocyte growth factor (28).

Although the immune processes taking place in the oral mucosa are still poorly understood, CD pathogenesis in the gut is very well characterized. IL-15, a cytokine structurally related to IL-2 that it is widely distributed, has been found to be upregulated in enterocytes in active CD. It drives epithelial damage by stimulating the production of Th1 cytokines, such as IFNγ, and the cytotoxicity of intestinal intraepithelial lymphocytes (IELs) (29–31). IL-15 contribution to oral manifestations in CD has not been addressed yet. Consistent with a gliadin-driven Th1 response, IELs in CD are enriched in cytolytic proteins, such as perforin, and produce large amounts of IFNγ; therefore, participating in the severe mucosal damage (32–34). Gluten-specific CD4+ T-cell lines and clones derived from CD patients were shown to produce interferon (IFN), in response to activated dendritic cells isolated from the mucosa of active CD (31, 35). There are a few studies describing oral intraepithelial dendritic cells (36, 37). These cells express langerin and are highly abundant in the oral mucosa (38). However, their role in CD has not been explored. Regarding the T-lymphocytic inflammatory infiltrate in the oral mucosa, it was found to be significantly increased in patients with active CD (6), in contrast to other studies that found no differences with healthy control (39, 40).

Previous studies by our group with allergic patients to grass pollen, olive pollen, or house dust mite (HDM) have demonstrated that they undergo oral epithelial remodeling characterized by reduced expression of claudin-1, occludin, and E-cadherin proteins (25, 41). In fact, severe grass pollen allergic patients also presented an increased number of CD11c+ and CD4+ infiltrates and increased gene expression of IL-33 (25). Moreover, these patients were characterized by unique transcriptomic and metabolomic fingerprints (42).

In view of these evidence, we formulated the hypothesis that oral mucosa remodeling could also be present in CD patients and would not only help us understand the extraintestinal manifestations associated to CD, but also provide a rationale for oral IT strategies in CD.

In this study, we have found that the oral epithelial barrier of CD patients is compromised, even when they adhere to a GFD. Moreover, increased Treg numbers in the oral mucosa are observed in CD patients. Our data suggest that the characterization of the oral mucosal barrier may be a potential tool for advancing novel oral diagnostic markers and disease modifying and tolerization treatments for CD.

## Materials and Methods

### Study Subjects

Twenty-one subjects were recruited: six CD patients *de novo* diagnosed, seven CD patients under GFD treatment for at least 1 year, and eight non-celiac subjects as a control group. *De novo* CD patients were included after confirmation of duodenal biopsy histological classification Marsh III (villi atrophy), positive Anti-Transglutaminase Antibodies (ATA), and DQ2+ allele of the Human Leukocyte Antigen (HLA). GFD treated group included patients with positive clinical response to diet and ATA loss. Medical history from all subjects was revised by the gastroenterologist in charge of patient enrolment. All subjects with a recent history of nasopharyngeal disease or severe respiratory allergic disease were excluded from the study. During sample collection, all patients were further investigated to ensure that no significant oral lesions were present. Complying with EU regulation 2016/679 from the European Parliament and the Council and Spanish Royal Decree-Law 5/2018, all subjects provided written informed consent and protocol approval was obtained from the Research and Ethics Committees of San Agustin Hospital of Aviles. Clinical features and detailed information of the studied population are shown in **Table 1**.

**Table 1:** Detailed information on study celiac disease patients.

	Age	Sex	Serology		Time since GFD	Genotype	Clinical manifestations	Other diseases	Marsh classification	Response to GFD
			*Total IgA(mg/dl)*	*IgA Anti-tTG2 (kU/L)*		*DQ2*				
**De novo**	42	F	143	158	–	+	ID	V	3b	
	64	F	80	12	–	+	ID, DY	–	3b	
	64	F	253	18.3	–	+	WL, N, D	HP	3b	
	25	F	<5*	26.1	–	+	ABP, ID, HC	SA, RD	3a	
	62	M	370	45.7	–	+	AN, GIB	HTN	3b	
	49	F	149	657	–	+	DY, D/CON	–	3a	
**GFD**	48	F	133	>125/5	6 y 9 m	+	DY, ID, AN	–	3c	+
	67	F	133	152/<5	20 y	+	D, WL	HT, FM, DE	3a	+
	26	F	152	>300/<5	1 y 1 m	+	ABP, WL	–	3b	+
	38	F	277	41.2/<5	12 y 5 m	+	AN, D	–	3b	+
	54	F	222	1.2	2y	+	ID, B12	VE, DE, AG	3a	+
	54	F	284	>300/9.8	1y 6 m	+	VO, D/CON, ABP	–	3b	+
	53	M	266	93/<5	1y 2 m	+	ABP	–	3b	+

M/F, male/female; y/m, years/months; AG, autoimmune gastritis; ABP, abdominal pain; AN, anemia; B12, B12 deficiency; CON, constipation; DE, depression; D, diarrhea; DY, dyspepsia; FM, fibromyalgia; GIB, gastrointestinal bleeding; HC, hematochezia; HP, Helicobacter pylori; HT, hypothyroidism; HTN, hypertension; ID, iron deficiency; N, nausea; RD, rheumatic disease; SA, sulfonamide allergy; V, vitiligo; VE, vertigo; VO, vomit; WL, weight loss.

### Duodenal Samples

Duodenal sampling was carried out under local anesthesia, obtaining a total of six biopsies from the second portion of the duodenum and the duodenal bulb. Endoscopy was carried out using a Fujifilm gastroscope series 200 or 500 with a biopsy channel of 2.8 mm. Biopsy specimens were fixed in 4% PFA, and later processed to paraffin blocks, sectioned at 4 µm and stained with H&E following manufacturer´s protocols. Duodenal damage was categorized according to Marsh classification (**Supplementary Figure 1**). Duodenal biopsies were performed at the moment of diagnosis, duodenal histopathological information for GFD treated patients was obtained from archived data.

### Serum Antitransglutaminase 2 Determinations

Serum antitransglutaminase 2 antibodies (IgA class) were measured using Triturus automated ELISA analyzer (Grifols). Total IgA was measured by immunoturbidimetry using a Cobas^©^ 8000 modular analyzer (Hoffman-La Roche).

### Oral Mucosa Samples

Two biopsies were taken from the buccal mucosa of each study subject using a 3 mm surgery biopsy punch under local anesthesia. One biopsy was embedded in 4% paraformaldehyde (PFA) and processed to paraffin for immunohistochemical (IHC) studies; and the other was conserved in RNALater™ (ThemoFisher) for qPCR studies.

### Blood Sampling

Twenty milliliters of heparinized blood were collected from each study subject. Ficoll-Paque (GE Healthcare™) density gradient centrifugation was performed to obtain both plasma and Peripheral Blood Mononuclear Cells (PBMCs). Plasma samples were frozen at −80°C for cytokine quantification by ELISA. PBMCs were further lysed in RLT buffer (Qiagen) and stored at −20°C for later RNA extraction.

### Oral Mucosa Immunohistochemical Analyses

Paraffin blocks were cut into 1 µm-thick sections and fixed onto a poly-L-lysine treated glass slide and then used for histological and immunohistochemical analyses. All sections were stained with H&E following manufacturer’s protocol to achieve correct orientation of the biopsy. Immunohistochemical staining was performed using Bond Polymer refine Detection kit on a BOND-MAX Automated IHC/ISH Stainer (Leica Biosystems) following the manufacturer’s protocol. The following antibodies were used: mouse monoclonal anti-human CD19 (NCL-L-CD19-163, Leica Biosystems), rat anti-human CD3 (MCA1477,Bio-Rad), mouse monoclonal anti-human CD4 (NCL-L-CD4-368, Leica Biosystems), mouse monoclonal anti-human CD8 (NCL-L-CD8-4B11, Leica Biosystems), mouse monoclonal anti-human Claudin-1 (ab56417, Abcam), mouse monoclonal anti-human E-Cadherin (36B5) (PA0387, Leica Biosystems), mouse monoclonal anti-human FoxP3 (ab22510, Abcam), mouse monoclonal anti-human γδ-TCR (sc-100289, Santa Cruz Biotechnology), mouse monoclonal anti-human Langerin (ab49730, Abcam), rabbit recombinant monoclonal [EPR20992] to Occludin (ab216327, Abcam), and rabbit polyclonal to Neutrophil Elastase (ab68672, Abcam). Positive and negative controls were included for each experiment.

### Luna Staining

Sections were stained with a mixture of hematoxylin and Biebrich scarlet for 5 min. Subsequently, they were rinsed with 1% acid alcohol solution (hydrochloric acid), and water. Finally, samples were counterstained with 0.5% lithium carbonate solution and rinsed in running water for 2 min.

### Oral Mucosa Image Analysis

Scanning of the samples was performed using pathology scanner Aperio Versa 8 (Leica Biosystems) at 40× magnification. A sufficient number of images covering the whole biopsy were captured at an appropriate magnification for quantification.

Epithelial cell junctions image analyses were performed using Image-Pro Plus v4.5.0.29 (Media Cybernetics). Briefly, an adequate threshold that best adjusted to the actual DAB staining was established for a selected area. After applying the same threshold to all the images taken from the same sample, the software calculated the stained area relative to the total selected area. The final value was calculated as the weighted average of all measurements obtained for each image in one sample. These analyses were done by at least two independent observers for each staining.

Inflammatory infiltrate analyses were performed by using the “counter tool” integrated in Aperio ImageScope v12.3.2.8013 (Leica Biosystems) covering either epithelium, connective tissue, or the whole sample, when appropriate. Both area and stained cells were determined by three independent observers.

### RNA Isolation From Oral Biopsies and Peripheral Blood Mononuclear Cells for qPCR

Oral mucosa tissue was digested in TRIzol™ (ThermoFisher) manually using a scalpel and later homogenized using TissueLyser (Qiagen). After centrifugation, aqueous phase was purified using RNeasy Mini Kit (Qiagen) columns with DNase treatment, according to manufacturer’s protocol, and later retrotranscribed into cDNA with the High Capacity RNA-to-cDNA kit (Applied Biosystems). RNA from lysed PBMCs was also extracted using RNeasy Mini Kit (Qiagen) columns. SYBR Green master mix (Takara) was used for quantitative RT-PCR in the equipment Real Time HT 7900 (Applied Biosystems).

Expression data were normalized to the average median of housekeeping genes β-actin and GAPDH and the results were analyzed using the 2 -ΔΔCT method (26). Oligonucleotides for selected genes were designed using Primer3 software, NIH PrimerBlast and Olygoanalizer tool (IDT).

### Plasma Cytokine Quantification

ELISA kits for IL-33 (CSB-E13000h-96T), IL-25 (CSB-E11715h-96T), and TSLP (CSB-E03316h-96T) (Cusabio) were used to determined cytokine plasma levels following manufacturer’s recommended protocols for every specific kit. To detect PAF in plasma, LabClinics ELISA kit (EH4331) was used. Optical density of the ELISAs was measured at 450 nm in a Varioskan plate reader. A five-parameter logistic fit curve was generated for each cytokine from the seven standards.

### Statistics

GraphPad Prism v8.0.1 software was used for statistical analysis. Non-parametric Kruskal-Wallis test followed by Dunn’s multiple comparison test or One-way ANOVA test followed by Tukey’s multiple comparison test were used when appropriate to compare data among experimental groups. For correlation analysis, Spearman correlation was applied. A p-value <0.05 was considered significant for all the analyses. Descriptive statistics along the text are expressed as “mean (s = standard deviation).” Inferential statistics are expressed as “(mean difference ± SE of difference, p-value)” when a parametric test is used, and “(median, p-value)” when the test is non-parametric.

## Results

### Clinical Features

Of the study subjects, 76.2% were female: 83.3% in the *de novo* group, 87.5% in the GFD group, and 62.5% in the control group. Average age was 44.69 years: 51 (s = 15.62 years) in the *de novo* group, 48.57 years (s = 13.16 years) in the GFD group, and 34.5 years (s = 10.81 years) in the control group. All included study subjects were Caucasian.

Among CD patients, average age of diagnosis was 46.6 years: 51 years (s = 15.62 years) for the *de novo* diagnosed and 42.21 years (s = 12.12 years) for the GFD group. The most frequent clinical manifestations at diagnosis were iron deficiency and diarrhea, found in 38.46% of the CD patients, being 23.08% diagnosed with anemia. Other frequent manifestations were abdominal pain (30.77%), weigh loss (23.08%), and dyspepsia (23.08%). Comorbidities were described in some patients and included other autoimmune diseases such as vitiligo or rheumatic disease. Of the patients in the GFD group, 25% showed signs of depression (**Table 1**).

According to the inclusion criteria, all newly diagnosed patients involved in the study tested positive for tissue transglutaminase 2 (tTG2) antibodies, genotype HLA-DQ2+ and reach at least III in Marsh scale for duodenal biopsy. All patients had normal IgA levels, except one that presented IgA deficiency, but clear anti-tTG2 antibodies. In the GFD group, average time under diet was 6.42 years (s = 6.76 years) and ranged between 1.17 and 12.42 years (**Table 1**).

### Epithelial Integrity of the Oral Mucosa Is Compromised in Celiac Disease Patients

**Figure 1:**
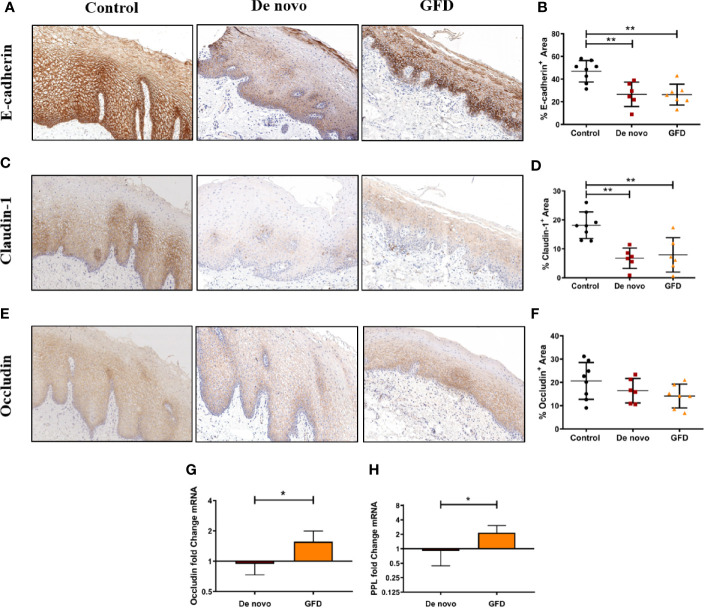
Immunohistochemical staining of FFPE oral mucosal sections and quantification of protein expression from non-celiac subjects (control) (n = 8) and CD patients *de novo* diagnosed (*de novo*) (n = 6) and after Gluten Free Diet (GFD) (n = 7) for E-cadherin **(A, B)**, claudin-1 **(C, D)**, and occludin **(E, F)**. Images were captured at 8× magnification. Quantification is the percentage‐stained area (mm^2^) of the total epithelial area **(B, D, F)**. Scatter plots show mean ± SD *p < 0.05, **p < 0.01. Fold change expression of occludin **(G)** and periplakin (PPL) **(H)** in the oral mucosa for *de novo* diagnosed (n = 6) and GFD (n = 7) CD patients. Fold change is referred to non-celiac (control) samples using 2 -ΔΔCT method. Data were normalized using two housekeeping genes (GAPDH and β-actin). Bar plots show mean ± SD *p < 0.05.

Protein expression levels of epithelial junctional proteins were studied by IHC staining in histological sections of the oral mucosa. Paraffin-embedded samples from all subjects included in the study were stained targeting occludin and claudin-1, members of the tight junctional complex, and E-cadherin, member of the adherens junctions. We found a significant decrease in E-cadherin expression in both groups of CD patients: *de novo* diagnosed (−20.34 ± 5.30%, p < 0.01) and GFD patients (−20.58 ± 5.08%, p < 0.01) compared with non-celiac controls (**Figures 1A, B**). Claudin-1 was also significantly decreased in both groups of CD patients when compared to control subjects (*de novo* diagnosed −12.97 ± 2.99%, p < 0.01 and GFD −11.81 ± 2.99%) (**Figures 1C, D**). For occludin, a non-significant trend was observed (**Figures 1E, F**).

We also determined mRNA expression of the epithelial junctional protein occludin, and periplakin (PPL), a member of desmosomes. We found increased mRNA expression in GFD patients for both occludin (+0.62 ± 0.20-fold change, p < 0.05) and PPL (+1.26 ± 0.41-fold change, p < 0.05) when compared to *de novo* patients (**Figures 1G, H**).

### Celiac Disease Patients Present Higher Treg Infiltrate in the Oral Epithelium

**Figure 2:**
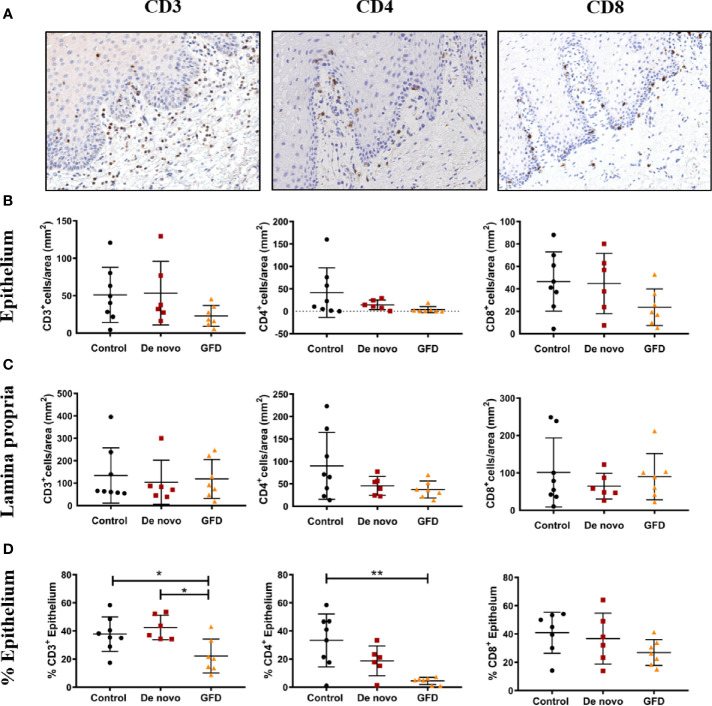
Representative images of CD3, CD4, CD8 immunohistochemistry **(A)**. Images were captured at 15× magnification. Absolute counts in epithelium **(B)**, absolute counts in lamina propria **(C)**, and frequency of cells present in the epithelium (%) **(D)** for CD3+ (left), CD4+ (center), and CD8+ (right) from non-celiac subjects (control) (n = 8) and celiac disease patients *de novo* diagnosed (*de novo*) (n = 6) and after Gluten Free Diet (GFD) (n = 7). Scatter plots show mean ± SD *p < 0.05, **p < 0.01.

Oral mucosa biopsies were stained for common T lymphocyte and antigen-presenting cell (APC) markers, such CD3, CD4, CD8, FoxP3, Langerin, and CD11c (**Figure 2** and **Supplementary Figure 2**). We found no significant differences in the absolute counts of CD3^+^, CD4^+^, or CD8^+^ T cells among the experimental groups in either epithelium or the lamina propria (**Figures 2B, C**). However, when the preferential location of these cells was examined, significant differences were found among study groups. The percentage of CD3^+^ cells located in the epithelium was significantly lower in GFD patients when compared to both control (−15.60 ± 5.86%, p < 0.05) and *de novo* subjects (−20.32 ± 6.30%, p < 0.05) (**Figure 2D**). CD4^+^ presence in the epithelium was also significantly lower in GFD patients but only when compared to control subjects (−28.91 ± 7.27%, p < 0.01) (**Figure 2D**). CD8^+^ cells showed no significant differences among experimental groups (**Figure 2D**). These lymphocytes lacked γδ T cell receptor expression (**Supplementary Figures 2A, E**).

**Figure 3:**
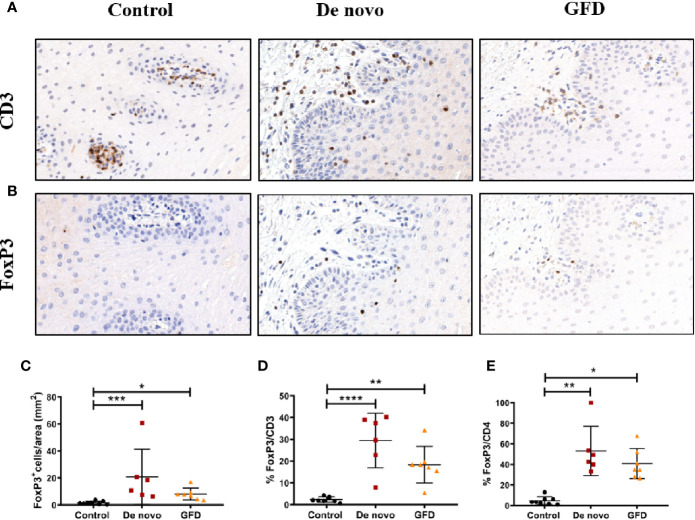
Representative images of CD3 **(A)** and FoxP3 **(B)** staining of oral mucosal sections. Images were captured at 20× magnification. Quantification of FoxP3+ cells in the whole mucosa **(C)** for non-celiac subjects (control) (n = 8) and celiac disease patients *de novo* diagnosed (*de novo*) (n = 6) and after Gluten Free Diet (GFD) (n = 7). Quantification is expressed as counts per area (mm^2^) of the total mucosa. Percentage of FoxP3+ cells in relation to CD3+ **(D)** or CD4+ **(E)** counting in the whole mucosa. Scatter plots show mean ± SD *p < 0.05, **p < 0.01, ***p < 0.001, ****p < 0.0001.

Fox p 3+ cell numbers in the epithelium were negligible in most cases (data not shown). FoxP3^+^ Treg counts in the whole mucosa were significantly higher in both *de novo* diagnosed (+11.54 cells/mm^2^, p < 0.001) and GFD (+8.04 cells/mm^2^, p < 0.05) CD patients as compared to the non-celiac (control) group (**Figures 3A, B**). When the population of Tregs among the total CD3^+^ and CD4^+^ cells was examined, similar results were observed. FoxP3^+^/CD4^+^ ratio was increased in *de novo* diagnosed (+11.00%, p < 0.01) and GFD (+9.00%, p < 0.05) groups. FoxP3^+^/CD3^+^ ratio followed the same pattern and was also increased in *de novo* (+27.11 ± 4.58%, p < 0.0001) and GFD (+16.01 ± 4.58%, p < 0.01) groups (**Figures 3C, D**).

We also stained for the APC markers langerin and CD11c to characterize whether there was a predominant APC population associated to CD. No significant differences were found in these two markers among the experimental groups (**Supplementary Figure 3**). Anti-CD19, anti-neutrophil elastase and Luna staining revealed absence of B cells, low presence of neutrophils, and absence of eosinophils, respectively, in buccal mucosa tissue (**Supplementary Figure 2**).

### Recruitment of Tregs to the Damaged Oral Mucosa Is Associated to the Upregulation of Amphiregulin

**Figure 4:**
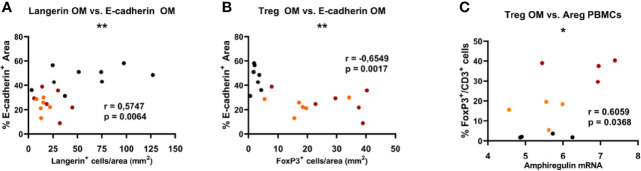
Significant Spearman correlations found in the oral mucosa between the expression of epithelial E-cadherin and langerin **(A)**, and FoxP3 Treg frequency **(B)**. Significant correlations found in oral mucosa and PBMCs between the frequency of FoxP3 Treg cells in the oral mucosa and the expression of amphiregulin (Areg) **(C)**. Red dots represent *de novo* diagnosed patients, orange dots represent GFD, and black dots correspond to non-celiac (control) subjects. *p < 0.05, **p < 0.01.

Next, we wanted to determine whether the recruitment of Tregs to the oral mucosa presented characteristic features. First, we found a significant positive correlation between E-cadherin expression and langerin expression in the oral epithelium (r = 0.57, p < 0.01) for all the experimental groups (**Figure 4A**). In contrast, E-cadherin levels negatively correlated to FoxP3^+^ Treg numbers (r = −0.65, p < 0.01), i.e. the higher the damage in the epithelium (the lower the E-cadherin expression), the higher the Treg infiltrate in the oral mucosa (**Figure 4B**). Moreover, Treg recruitment to the oral mucosa was found to be positively correlated with amphiregulin mRNA expression in PBMCs (r = 0.61, p < 0.05) (**Figure 4C**).

### Markers Associated With Celiac Disease in the Intestinal Mucosa Are Not Increased in the Oral Mucosa

**Figure 5:**
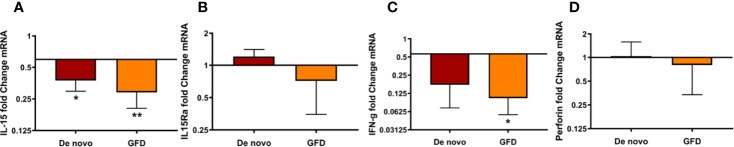
mRNA expression of *de novo* diagnosed CD patients (*de novo*) (n = 6) and CD patients on GFD (GFD) (n = 7) for IL-15 **(A)**, IL15Ra **(B)**, IFNγ **(C),** and perforin **(D)**. Fold change is referred to non-celiac (control) samples using 2 -ΔΔCT method, data were normalized using two housekeeping genes (GAPDH and β-actin). Bar plots show mean ± SD *p < 0.05, **p <0.01.

Next, we studied whether factors traditionally associated with intestinal IELs and inflammation in CD were also relevant in the oral mucosa of CD patients. For that, we determined IL15, IL15RA, IFNγ, and perforin mRNA levels in oral mucosa biopsies. Surprisingly, we found that all tended to decrease in CD patients, being this decrease significant for IL-15 in *de novo* diagnosed (0.55 ± 0.20 fold change, p < 0.05) and GFD (0.71 ± 0.19 fold change, p < 0.01) patients, and IFNγ expression in GFD patients (1.51 ± 0.55 fold change, p < 0.05) when compared to the control group (**Figure 5**).

### IL33 Plasma Levels Are Increased in Celiac Disease Patients

**Figure 6:**
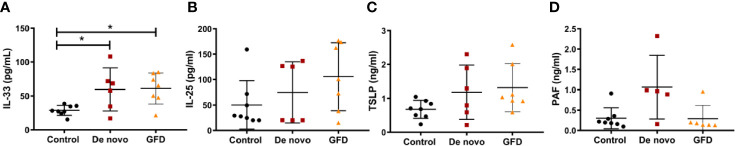
Protein levels of IL-33 **(A),** IL-25 **(B),** TSLP **(C),** and PAF **(D)** in plasma samples of non-celiac subjects (control) (n = 8), *de novo* diagnosed CD patients (*de novo*) (n = 6), and CD patients on GFD (GFD) (n = 7). Scatter plots show mean ± SD. *p-value <0.05.

Levels of the epithelial alarmins known to be released upon mucosal damage were determined in oral mucosa biopsies and in plasma samples of the study subjects. In oral mucosa biopsies, we did not find significant differences in mRNA expression for IL-33, TSLP, or periostin (*POSTN*) of CD patients (**Supplementary Figure 4**). Plasma levels of IL-33, IL-25, and TSLP were measured by ELISA. D*e novo* diagnosed (26.78 ± 10.21 pg/ml, p < 0.05) and GFD (27.53 ± 9.30 pg/ml, p < 0.05) CD patients presented significantly higher plasma levels of IL-33 as compared with non-celiac subjects (**Figure 6A**). There were no significant differences between the two groups of CD patients. We could not find any differences among the experimental groups for IL-25 or TSLP, although a trend of higher TSLP plasma levels was observed for both groups of CD patients when compared to controls (p = 0.059) (**Figures 6B, C**). We also determined PAF levels, as it is known to induce IL-33 release. We could not find any differences between non-celiac and either of the CD groups (p = 0.09). Nevertheless, PAF plasma levels tended to be higher in *de novo* diagnosed CD patients when compared to GFD (p = 0.09) (**Figure 6D**).

## Discussion

Celiac disease is a chronic autoimmune enteropathy triggered by gluten intake. As such, immune responses in the intestinal mucosa have been profoundly investigated (1, 43). However, it is well known that CD may present in atypical forms including extra-intestinal manifestations, some affecting the oral mucosa (6, 7). In fact, the oral mucosa of CD patients was shown to react to gliadin challenge with increasing numbers of lymphocytes (44). However, studies examining oral histopathological findings in CD are conflicting (8). In the present study, we examined buccal mucosa biopsies from CD patients both *de novo* diagnosed and under GFD, with the aim of determining its potential role as an immunomodulatory site for CD IT.

An intact functional oral mucosal barrier is crucial in the maintenance of homeostasis as it protects the mucosal immune system from the exposure to noxious environmental antigens (45). In our study, we have found that the expression of intercellular junctional proteins that are critical for epithelial integrity was altered in the buccal epithelium of CD patients even after avoidance of gluten for at least 1 year. In accordance, damage in the oral mucosa of GFD patients has been previously reported (6, 46). Unlike the previous studies that have focused on the characterization of the oral immune response, we have also examined the expression of intercellular junctional proteins to describe the remodeling of the oral mucosal barrier. This remodeling in the treated CD patients has been suggested not to result from poor dietary compliance, but rather as a late immune response reflecting chronic immune stimulation followed by regeneration of memory T cells (46). After gluten avoidance, mRNA levels of both occludin and PPL (a protein expressed in desmosomes and described to be a regulator of lung injury and repair) (47) are increased. These findings suggest that gluten depletion has a healing effect over the oral mucosa that is still not visible at the protein level. Therefore, an impaired epithelial barrier could account for the extraintestinal manifestations of CD observed in the oral cavity such as aphthous ulcers, even after the avoidance of gluten (48). In our study, all subjects were investigated by the gastroenterologist to ensure that no significant oral lesions were present in the buccal mucosa before enrolment. However, the overall health status of the oral cavity was not examined. Therefore, other alterations, e.g. gingivitis or periodontitis, cannot be formally excluded, although none of the study subjects showed signs of oral disease at sampling.

Regarding the immune infiltrate in the oral mucosa of CD patients, there are not consensus studies (6, 8, 39, 40). In our study, we did not find significant differences in the global counts of langerin^+^, CD11c^+^, CD4^+^, CD8^+^, or CD3^+^ cell populations. The study by Bardellini et al. describes an increased CD3 infiltrate in the stromal papillae that is decreased after avoidance of gluten (6). Although we could not see changes in absolute cell counts, we found that avoidance of gluten reduces the relative abundance of lymphocytes in the epithelium. Moreover, we also confirm that the vast majority of the IELs in the oral mucosa lack γδ-T cell receptor expression, in accordance with Lähteenoja et al. (46). These authors suggest that NK cells substantially contribute to lymphocyte recruitment; thus, NK cells may take part in the mucosa remodeling process. Although important in gut mucosa, we discard γδ-T cells as a biomarker in the epithelium of buccal mucosa of CD patients. In contrast, Krishnan S et al. found the presence of γδ+ cells at the gingiva in a mouse model of periodontitis. Moreover, these cells were producing amphiregulin for safeguarding the homeostasis of the oral mucosal barrier (49).

Interestingly, the population of Tregs was greatly increased in the oral mucosa of CD patients. However, previous research in circulating Tregs of CD patients suggests that they have an impaired suppressive function (50, 51). IL15, an important hallmark of CD, has been shown to perform a relevant role in Treg effect suppression (29, 52, 53). In the present study, we could not find an increase in IL-15 expression in the oral mucosa of CD patients. What we found is that Fox p3+ cell abundance inversely correlated with E-cadherin expression (i.e. the lower the E-cadherin expression, the more the Treg numbers in the buccal mucosa). Thus, we hypothesize that the Fox p3+ cells we observed in CD patients are recruited to protect against further tissue damage and maintain barrier integrity, as previously reported (28, 41). In fact, we found a positive correlation between Fox p3+ cell numbers in the oral mucosa and peripheral amphiregulin expression, which is a repair factor. Peripheral amphiregulin expression has been previously found associated to repair/remodeling features in other disease settings such as infant viral bronchiolitis (54). In this line, amphiregulin-producing pathogenic memory Th2 cells were found to control airway fibrosis resulting from chronic inflammatory stimulation (55). Besides, amphiregulin is described to restore integrity of damaged intestinal mucosa in murine models of acute graft-*versus*-host disease (aGVHD). In the study by Holtan S et al., high circulating amphiregulin levels reclassified patients into high risk subgroups helping further refine the aGVHD clinical risk score (56). Therefore, we propose that circulating amphiregulin could also be useful for the diagnosis of CD and helpful to establish an alternative classification score to the intestinal biopsy-based Marsh scoring system. Nevertheless, amphiregulin determination and Treg phenotypes in the oral mucosa deserve further study in the context of CD for solid conclusions.

We did not identify changes in langerin+ or CD11c+ cell counts in our study. However, langerin+ cells, described to have a tolerogenic role in the oral mucosa (57), correlated inversely with epithelial damage. This result supports the role of langerin+ cells as tolerance inductors.

Plasma cells have been recently identified as the most abundant gluten peptide MHC-expressing cells in the intestine of patients with active CD (58). Therefore, we investigated whether B cells were also more abundant in the oral mucosa of CD patients. Strikingly, our study revealed absence of B cells in the oral mucosa of CD patients. In addition and consistent with previous findings for allergic patients (25, 41), eosinophilic and neutrophilic infiltrates were negligible in the oral mucosa. According to Moutsopoulos et al., neutrophils are the gatekeepers of oral immunity. They can be found within the oral cavity, exhibiting varying levels of activation and functionality depending on the presence of oral inflammation. In patients with neutrophil defects, a dysregulated IL-17/Th17 response has been shown to drive immunopathology (19). Regarding eosinophils, there is limited evidence that they reside in the oral cavity, at least in the gingiva, in healthy individuals (19).

The immunomodulatory mechanisms taking place in the oral mucosa in CD support its potential role as a target for oral IT. The potential of the buccal mucosa as a drug delivery system has been previously reviewed (59) and trials have been made to develop an IT for CD (12, 60). Protein-based desensitization IT is widely used to treat allergic diseases (61). CD4+ Tregs are induced by peptide vaccination, which means that a sustainable induction of Tregs is responsible for the efficacy of this treatment (16, 62, 63). An important question we wanted to address was whether the inflammatory mechanisms taking place in the oral mucosa mirror those of the intestinal mucosa. In this regard, two studies have assessed the capacity of the oral mucosa of untreated CD patients to produce CD autoantibodies (64, 65). In our study, we assessed the expression of inflammatory factors such as IL-15, in the oral mucosa of CD patients. Surprisingly, IL-15 expression, along with that of IFNγ and perforin, was decreased. Therefore, the immunopathological mechanisms taking place in the oral mucosa of CD patients deserve further study.

In our study, the structural changes in the oral mucosa of CD patients take place with increased number of FoxP3 Tregs. Moreover, the inflammatory hallmark of CD in the gut (IL-15) seems to be absent in the oral mucosa. Furthermore, IL-33, TSLP, or POSTN mRNA levels are not elevated in buccal biopsies. Thus, we hypothesize that the oral mucosa remodeling observed may be a consequence of the systemic inflammation associated to CD. In fact, systemic immune deregulation is reflected in the function of the oral immune system (45). We, and others (66) have reported increased plasma levels of IL-33 in CD groups. Lopez-Casado et al. measured serum levels and determined intestinal expression of IL-33 and its receptor ST2 in patients with active CD, but not in patients following a GFD. They found that the higher levels of IL-33 and its receptor ST2 in the intestine and serum reflect an active inflammatory state. Thus, they suggest it may be a potential biomarker for CD. In this line, Perez F et al. (67), found an increased expression of IL-33 in the duodenal mucosa of active CD patients. These findings highlight the potential contribution of IL-33 to exacerbate inflammation in CD pathology.

Based on our results, we conclude that oral mucosal integrity is compromised in CD patients, even after gluten avoidance. A remodeled epithelium may be key for the IT to gain access to both epithelial surfaces (apical and basolateral) and the local mucosa associated immune system, as previously suggested (25, 68, 69). Therefore, a disrupted epithelial barrier, together with the local recruitment of Tregs, make the oral mucosa a potential target for CD IT. These features could also help explain oral extraintestinal manifestations, and possibly assist CD diagnosis. Overall, our study highlights the relevance to characterize the specific immunopathological features of the oral mucosa in CD.

## Data Availability Statement

The original contributions presented in the study are included in the article/**Supplementary Material**. Further inquiries can be directed to the corresponding author.

## Ethics Statement

The studies involving human participants were reviewed and approved by the Research and Ethics Committees of San Agustin Hospital of Aviles. The patients/participants provided their written informed consent to participate in this study.

## Author Contributions

LS, CDF, and JSS recruited the study subjects and conducted the biopsy and blood sampling. JSS, CPT, and CGC performed and analyzed the laboratory experiments. JSS, PS, DB, and CGC discussed the results and wrote the manuscript. All the authors contributed to the article and approved the submitted version.

## Funding

This study was financed by Allero Therapeutics BV, Rotterdam. JSS was supported by an FPI‐CEU predoctoral fellowship. CGC was supported by a postdoctoral contract co-funded by the competitive Program “Attracting Talent,” Community of Madrid, Spain.

## Conflict of Interest

PS is a shareholder of Allero Therapeutics.

The remaining authors declare that the research was conducted in the absence of any commercial or financial relationships that could be construed as a potential conflict of interest.

## References

[1] Sollid LM. Coeliac disease: dissecting a complex inflammatory disorder. Nat Rev Immunol (2002) 2(9):647–55. 10.1038/nri885 12209133

[2] Jabri B Sollid LM. Mechanisms of Disease: immunopathogenesis of celiac disease. Nat Clin Pract Gastroenterol Hepatol (2006) 3(9):516–25. 10.1038/ncpgasthep0582 16951668

[3] Green PHR Cellier C. Celiac Disease. N Engl J Med (2007) 357(17):1731–43. 10.1056/NEJMra071600 17960014

[4] Gujral N. Celiac disease: Prevalence, diagnosis, pathogenesis and treatment. World J Gastroenterol (2012) 18(42):6036. 10.3748/wjg.v18.i42.6036 23155333 PMC3496881

[5] Marsh MN. Gluten, major histocompatibility complex, and the small intestine. A molecular and immunobiologic approach to the spectrum of gluten sensitivity (‘celiac sprue’). Gastroenterology (1992) 102(1):330–54. 10.1016/0016-5085(92)91819-P 1727768

[6] Bardellini E Amadori F Ravelli A Salemme M Lonardi S Villanacci V. Histopathological findings in the oral mucosa of celiac patients. Rev Española Enfermedades Dig (2014) 106(2):86–91. 10.4321/S1130-01082014000200003 24852733

[7] Rodrigo L Beteta-Gorriti V Alvarez N Gómez de Castro C de Dios A Palacios L. Cutaneous and Mucosal Manifestations Associated with Celiac Disease. Nutrients (2018) 10(7):800. 10.3390/nu10070800 PMC6073559 29933630

[8] Bernardo D. Coeliac disease in the oral mucosa? Rev Española Enfermedades Dig (2014) 106(2):73–6. 10.4321/S1130-01082014000200001 24852731

[9] Shah S Akbari M Vanga R Kelly CP Hansen J Theethira T. Patient Perception of Treatment Burden Is High in Celiac Disease Compared With Other Common Conditions. Am J Gastroenterol (2014) 109(9):1304–11. 10.1038/ajg.2014.29 PMC4159418 24980880

[10] Singh J Whelan K. Limited availability and higher cost of gluten-free foods. J Hum Nutr Diet (2011) 24(5):479–86. 10.1111/j.1365-277X.2011.01160.x 21605198

[11] Niland B Cash BD. Health Benefits and Adverse Effects of a Gluten-Free Diet in Non-Celiac Disease Patients. Gastroenterol Hepatol (N Y) (2018) 14(2):82–91. 29606920 PMC5866307

[12] Di Sabatino A Lenti MV Corazza GR Gianfrani C. Vaccine Immunotherapy for Celiac Disease. Front Med (2018) 5:187. 10.3389/fmed.2018.00187 PMC6028606 29998106

[13] Palomares O Akdis M Martín-Fontecha M Akdis CA. Mechanisms of immune regulation in allergic diseases: the role of regulatory T and B cells. Immunol Rev (2017) 278(1):219–36. 10.1111/imr.12555 28658547

[14] Akdis M Akdis CA. Mechanisms of allergen-specific immunotherapy: Multiple suppressor factors at work in immune tolerance to allergens. J Allergy Clin Immunol (2014) 133(3):621–31. 10.1016/j.jaci.2013.12.1088 24581429

[15] Durham SR Emminger W Kapp A de Monchy JGR Rak S Scadding GK. SQ-standardized sublingual grass immunotherapy: Confirmation of disease modification 2 years after 3 years of treatment in a randomized trial. J Allergy Clin Immunol (2012) 129(3):717–725.e5. 10.1016/j.jaci.2011.12.973 22285278

[16] Varona R Ramos T Escribese MM Jimeno L Galán A Würtzen PA. Persistent regulatory T-cell response 2 years after 3 years of grass tablet SLIT: Links to reduced eosinophil counts, sIgE levels, and clinical benefit. Allergy (2019) 74(2):349–60. 10.1111/all.13553 PMC6585999 30003552

[17] Barber D Rico P Blanco C Fernandez-Rivas M Ibañez MD Escribese MM. GRAZAX®: a sublingual immunotherapy vaccine for Hay fever treatment: from concept to commercialization. Hum Vaccin Immunother (2019) 15(12):2887–95. 10.1080/21645515.2019.1622976 PMC6930101 31157592

[18] Kitamoto S Nagao-Kitamoto H Hein R Schmidt TM Kamada N. The Bacterial Connection between the Oral Cavity and the Gut Diseases. J Dent Res (2020) 99(9):1021–9. 10.1177/0022034520924633 PMC7375741 32464078

[19] Moutsopoulos NM Konkel JE. Tissue-Specific Immunity at the Oral Mucosal Barrier. Trends Immunol (2018) 39(4):276–87. 10.1016/j.it.2017.08.005 PMC5843496 28923364

[20] Shin K Fogg VC Margolis B. Tight Junctions and Cell Polarity. Annu Rev Cell Dev Biol (2006) 22(1):207–35. 10.1146/annurev.cellbio.22.010305.104219 16771626

[21] Leoni G Neumann P-A Sumagin R Denning TL Nusrat A. Wound repair: role of immune–epithelial interactions. Mucosal Immunol (2015) 8(5):959–68. 10.1038/mi.2015.63 PMC4916915 26174765

[22] Novak N Haberstok J Bieber T Allam J-P. The immune privilege of the oral mucosa. Trends Mol Med (2008) 14(5):191–8. 10.1016/j.molmed.2008.03.001 18396104

[23] Murota H Lingli Y Katayama I. Periostin in the pathogenesis of skin diseases. Cell Mol Life Sci (2017) 74:4321–8. 10.1007/s00018-017-2647-1 PMC11107733 28916993

[24] Khurshid Z Mali M Adanir N Zafar MS Khan RS Latif M. Periostin: Immunomodulatory Effects on Oral Diseases. Eur J Dent (2020) 14(3):462–6. 10.1055/s-0040-1714037 PMC7440953 32688410

[25] Rosace D Gomez-Casado C Fernandez P Perez-Gordo M Dominguez M del C Vega A. Profilin-mediated food-induced allergic reactions are associated with oral epithelial remodeling. J Allergy Clin Immunol (2019) 143(2):681–90.e1. 10.1016/j.jaci.2018.03.013 29705246

[26] Gomez-Casado C Villaseñor A Rodriguez-Nogales A Bueno J Barber D Escribese M. Understanding Platelets in Infectious and Allergic Lung Diseases. Int J Mol Sci (2019) 20(7):1730. 10.3390/ijms20071730 PMC6480134 30965568

[27] Morita H Arae K Unno H Miyauchi K Toyama S Nambu A. An Interleukin-33-Mast Cell-Interleukin-2 Axis Suppresses Papain-Induced Allergic Inflammation by Promoting Regulatory T Cell Numbers. Immunity (2015) 43(1):175–86. 10.1016/j.immuni.2015.06.021 PMC4533925 26200013

[28] Zhang C Li L Feng K Fan D Xue W Lu J. ‘Repair’ Treg Cells in Tissue Injury. Cell Physiol Biochem (2017) 43(6):2155–69. 10.1159/000484295 29069643

[29] Abadie V Jabri B. IL-15: a central regulator of celiac disease immunopathology. Immunol Rev (2014) 260(1):221–34. 10.1111/imr.12191 PMC4066219 24942692

[30] Di Sabatino A Ciccocioppo R Cupelli F Cinque B Millimaggi D Clarkson MM. Epithelium derived interleukin 15 regulates intraepithelial lymphocyte Th1 cytokine production, cytotoxicity, and survival in coeliac disease. Gut (2006) 55(4):469–77. 10.1136/gut.2005.068684 PMC1856172 16105889

[31] Meresse B Ripoche J Heyman M Cerf-Bensussan N. Celiac disease: from oral tolerance to intestinal inflammation, autoimmunity and lymphomagenesis. Mucosal Immunol (2009) 2(1):8–23. 10.1038/mi.2008.75 19079330

[32] Nilsen EM Lundin KE Krajci P Scott H Sollid LM Brandtzaeg P. Gluten specific, HLA-DQ restricted T cells from coeliac mucosa produce cytokines with Th1 or Th0 profile dominated by interferon gamma. Gut (1995) 37(6):766–76. 10.1136/gut.37.6.766 PMC1382937 8537046

[33] Guy-Grand D DiSanto JP Henchoz P Malassis-Séris M Vassalli P. Small bowel enteropathy: role of intraepithelial lymphocytes and of cytokines (IL-12, IFN-gamma, TNF) in the induction of epithelial cell death and renewal. Eur J Immunol (1998) 28(2):730–44. 10.1002/(SICI)1521-4141(199802)28:02<730::AID-IMMU730>3.0.CO;2-U 9521083

[34] Olaussen RW Johansen FE Lundin KEA Jahnsen J Brandtzaeg P Farstad IN. Interferon-γ-Secreting T Cells Localize to the Epithelium in Coeliac Disease. Scand J Immunol (2002) 56(6):652–64. 10.1046/j.1365-3083.2002.01195.x 12472679

[35] Ráki M Tollefsen S Molberg Ø Lundin KEA Sollid LM Jahnsen FL. A Unique Dendritic Cell Subset Accumulates in the Celiac Lesion and Efficiently Activates Gluten-Reactive T Cells. Gastroenterology (2006) 131(2):428–38. 10.1053/j.gastro.2006.06.002 16890596

[36] Hovav A-H. Dendritic cells of the oral mucosa. Mucosal Immunol (2014) 7(1):27–37. 10.1038/mi.2013.42 23757304

[37] Cutler CW Jotwani R. Dendritic Cells at the Oral Mucosal Interface. J Dent Res (2006) 85(8):678–89. 10.1177/154405910608500801 PMC2254185 16861283

[38] Incorvaia C Frati F Sensi L Riario-Sforza G Marcucci F. Allergic Inflammation and the Oral Mucosa. Recent Pat Inflammation Allergy Drug Discovery (2007) 1(1):35–8. 10.2174/187221307779815129 19075964

[39] Compilato D Campisi G Pastore L Carroccio A. The Production of the Oral Mucosa of Antiendomysial and Anti—Tissue-Transglutaminase Antibodies in Patients with Celiac Disease: A Review. Sci World J (2010) 10:2385–94. 10.1100/tsw.2010.228 PMC5763970 21170489

[40] Campisi G Compilato D Iacono G Maresi E Di Liberto C Di Marco V. Histomorphology of healthy oral mucosa in untreated celiac patients: unexpected association with spongiosis. J Oral Pathol Med (2008) 38(1):34–41. 10.1111/j.1600-0714.2008.00677.x 18673416

[41] Sanchez-Solares J Delgado-Dolset MI Mera-Berriatua L Hormias-Martin G Cumplido JA Saiz V. Respiratory allergies with no associated food allergy disrupt oral mucosa integrity. Allergy (2019) 74(11):2261–5. 10.1111/all.13860 31077403

[42] Obeso D Mera-Berriatua L Rodríguez-Coira J Rosace D Fernández P Martín-Antoniano IA. Multi-omics analysis points to altered platelet functions in severe food-associated respiratory allergy. Allergy (2018) 73(11):2137–49. 10.1111/all.13563 30028518

[43] Iversen R Sollid LM. Autoimmunity provoked by foreign antigens. Sci (80 ) (2020) 368(6487):132–3. 10.1126/science.aay3037 32273455

[44] Lahteenoja H Maki M Viander M Toivanen A Syrjanen S. Local challenge of oral mucosa with gliadin in patients with coeliac disease. Clin Exp Immunol (2000) 120(1):38–45. 10.1046/j.1365-2249.2000.01177.x 10759761 PMC1905618

[45] Moutsopoulos N Moutsopoulos H. The oral mucosa: A barrier site participating in tissue-specific and systemic immunity. Oral Dis (2018) 24(1–2):22–5. 10.1111/odi.12729 29480644

[46] Lahteenoja H Toivanen A Viander M Raiha I Rantala I Syrjanen S. Increase in T-Cell Subsets of Oral Mucosa: a Late Immune Response in Patients with Treated Coeliac Disease? Scand J Immunol (2000) 52(6):602–8. 10.1046/j.1365-3083.2000.00794.x 11119267

[47] Besnard V Dagher R Madjer T Joannes A Jaillet M Kolb M. Identification of periplakin as a major regulator of lung injury and repair in mice. JCI Insight (2018) 3(5):e90163. 10.1172/jci.insight.90163 PMC5922284 29515024

[48] Rashid M Zarkadas M Anca A Limeback H. Oral manifestations of celiac disease: a clinical guide for dentists. J Can Dent Assoc (2011) 77:b39. 21507289

[49] Krishnan S Prise IE Wemyss K Schenck LP Bridgeman HM McClure FA. Amphiregulin-producing γδ T cells are vital for safeguarding oral barrier immune homeostasis. Proc Natl Acad Sci U S A (2018) 115(42):10738–43. 10.1073/pnas.1802320115 PMC6196490 30279177

[50] Cook L Munier CML Seddiki N van Bockel D Ontiveros N Hardy MY. Circulating gluten-specific FOXP3 + CD39 + regulatory T cells have impaired suppressive function in patients with celiac disease. J Allergy Clin Immunol (2017) 140(6):1592–603.e8. 10.1016/j.jaci.2017.02.015 28283419

[51] Granzotto M dal Bo S Quaglia S Tommasini A Piscianz E Valencic E. Regulatory T-Cell Function Is Impaired in Celiac Disease. Dig Dis Sci (2009) 54(7):1513–9. 10.1007/s10620-008-0501-x 18975083

[52] Jabri B Sollid LM. T Cells in Celiac Disease. J Immunol (2017) 198(8):3005–14. 10.4049/jimmunol.1601693 PMC5426360 28373482

[53] Zanzi D Stefanile R Santagata S Iaffaldano L Iaquinto G Giardullo N. IL-15 Interferes With Suppressive Activity of Intestinal Regulatory T Cells Expanded in Celiac Disease. Am J Gastroenterol (2011) 106(7):1308–17. 10.1038/ajg.2011.80 21468011

[54] Jones AC Anderson D Galbraith S Fantino E Cardenas DG Read JF. Personalized transcriptomics reveals heterogeneous immunophenotypes in children with viral bronchiolitis. Am J Respir Crit Care Med (2019) 199(12):1537–49. 10.1164/rccm.201804-0715OC 30562046

[55] Hirahara K Aoki A Morimoto Y Kiuchi M Okano M Nakayama T. The immunopathology of lung fibrosis: amphiregulin-producing pathogenic memory T helper-2 cells control the airway fibrotic responses by inducing eosinophils to secrete osteopontin. Semin Immunopathol (2019) 41:339–48. 10.1007/s00281-019-00735-6 30968186

[56] Holtan SG DeFor TE Panoskaltsis-Mortari A Khera N Levine JE Flowers MED. Amphiregulin modifies the Minnesota acute graft-versus-host disease risk score: Results from BMT CTN 0302/0802. Blood Adv (2018) 2(15):1882–8. 10.1182/bloodadvances.2018017343 PMC6093743 30087106

[57] Upadhyay R Jaitley S Shekhar R Agrawal P Upadhyay J. Langerhans cells and their role in oral mucosal diseases. N Am J Med Sci (2013) 5(9):505. 10.4103/1947-2714.118923 24251267 PMC3818822

[58] Høydahl LS Richter L Frick R Snir O Gunnarsen KS Landsverk OJB. Plasma Cells Are the Most Abundant Gluten Peptide MHC-expressing Cells in Inflamed Intestinal Tissues From Patients With Celiac Disease. Gastroenterology (2019) 156(5):1428–39.e10. 10.1053/j.gastro.2018.12.013 30593798 PMC6441630

[59] Montenegro-Nicolini M Morales JO. Overview and Future Potential of Buccal Mucoadhesive Films as Drug Delivery Systems for Biologics. AAPS Pharm Sci Tech (2017) 18:3–14. 10.1208/s12249-016-0525-z 27084567

[60] Sollid LM Khosla C. Novel therapies for coeliac disease. J Intern Med (2011) 269(6):604–13. 10.1111/j.1365-2796.2011.02376.x PMC3101315 21401739

[61] Larché M Wraith DC. Peptide-based therapeutic vaccines for allergic and autoimmune diseases. Nat Med (2005) 11(S4):S69–76. 10.1038/nm1226 15812493

[62] Verhoef A Alexander C Kay AB Larché M. T Cell Epitope Immunotherapy Induces a CD4+ T Cell Population with Regulatory Activity. Platts-Mills T, editor. PloS Med (2005) 2(3):e78. 10.1371/journal.pmed.0020078 15783262 PMC1069669

[63] Tanaka Y Fukumoto S Sugawara S. Mechanisms underlying the induction of regulatory T cells by sublingual immunotherapy. J Oral Biosci (2019) 61(2):73–7. 10.1016/j.job.2019.02.001 31109864

[64] Carroccio A Campisi G Iacono G Iacono OL Maresi E Di Prima L. Oral mucosa of coeliac disease patients produces antiendomysial and antitransglutaminase antibodies: the diagnostic usefulness of an in vitro culture system. Aliment Pharmacol Ther (2007) 25(12):1471–7. 10.1111/j.1365-2036.2007.03335.x 17539987

[65] Vetrano S Zampaletta U Anania MC Di Tola M Sabbatella L Passarelli F. Detection of anti-endomysial and anti-tissue transglutaminase autoantibodies in media following culture of oral biopsies from patients with untreated coeliac disease. Dig Liver Dis (2007) 39(10):911–6. 10.1016/j.dld.2007.07.158 17719860

[66] López-Casado MA Lorite P Palomeque T Torres MI. Potential role of the IL-33/ST2 axis in celiac disease. Cell Mol Immunol (2017) 14(3):285–92. 10.1038/cmi.2015.85 PMC5360881 26343805

[67] Perez F Ruera CN Miculan E Carasi P Dubois-Camacho K Garbi L. IL-33 Alarmin and Its Active Proinflammatory Fragments Are Released in Small Intestine in Celiac Disease. Front Immunol (2020) 11:581445. 10.3389/fimmu.2020.581445 33133101 PMC7578377

[68] Sanchez-Solares J Delgado-Dolset MI Mera-Berriatua L Hormias-Martin G Cumplido JA Saiz V. Respiratory allergies with no associated food allergy disrupt oral mucosa integrity. Allergy: Eur J Allergy Clin Immunol (2019). 10.1111/all.13860 31077403

[69] Rybakovsky E Valenzano MC Deis R Diguilio KM Thomas S Mullin JM. Improvement of Human-Oral-Epithelial-Barrier Function and of Tight Junctions by Micronutrients. J Agric Food Chem (2017). 10.1021/acs.jafc.7b04203 29172516

