# Supplementary material for: Celiac Disease Causes Epithelial Disruption and Regulatory T Cell Recruitment in the Oral Mucosa
Source: Front Immunol. 2021 Feb 25;12:623805. doi: 10.3389/fimmu.2021.623805 (PMC7947325; doi:10.3389/fimmu.2021.623805)
Supplement: Supplementary file 1 [file DataSheet_1.docx]

**Supplementary material**

**Supplementary Figure 1**

**Supplementary Figure 2**

**Supplementary Figure 3**

**Supplementary Figure 4**

**
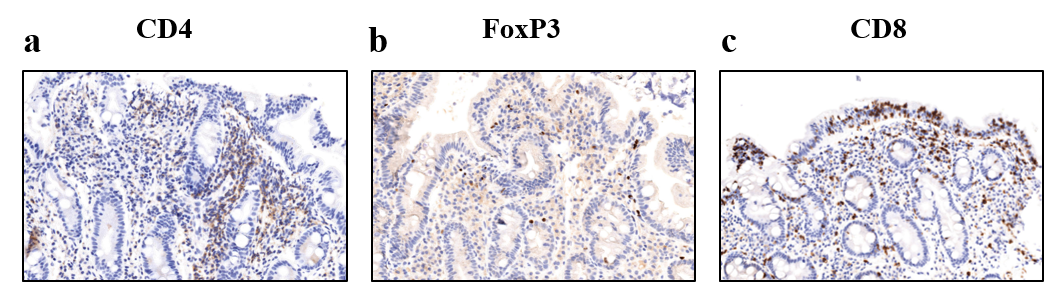
**

**Figure S1: Immunohistochemistry of duodenal biopsies**

Representative images of immunohistochemistry of duodenal biopsies from de novo CD diagnosed patients. Samples show significant villi atrophy, crypt hyperplasia and IEL infiltration compatible with stage III of the Marsh classification score. **(a)** Abundant CD4+ infiltration in the submucosa and lamina propria is shown**. (b)** FoxP3+ population is present in duodenal biopsies. **(c)** CD8+ cytotoxic T cell population is observed in the epithelium, a hallmark of CD inflammation. Images were captured at 10X magnification.


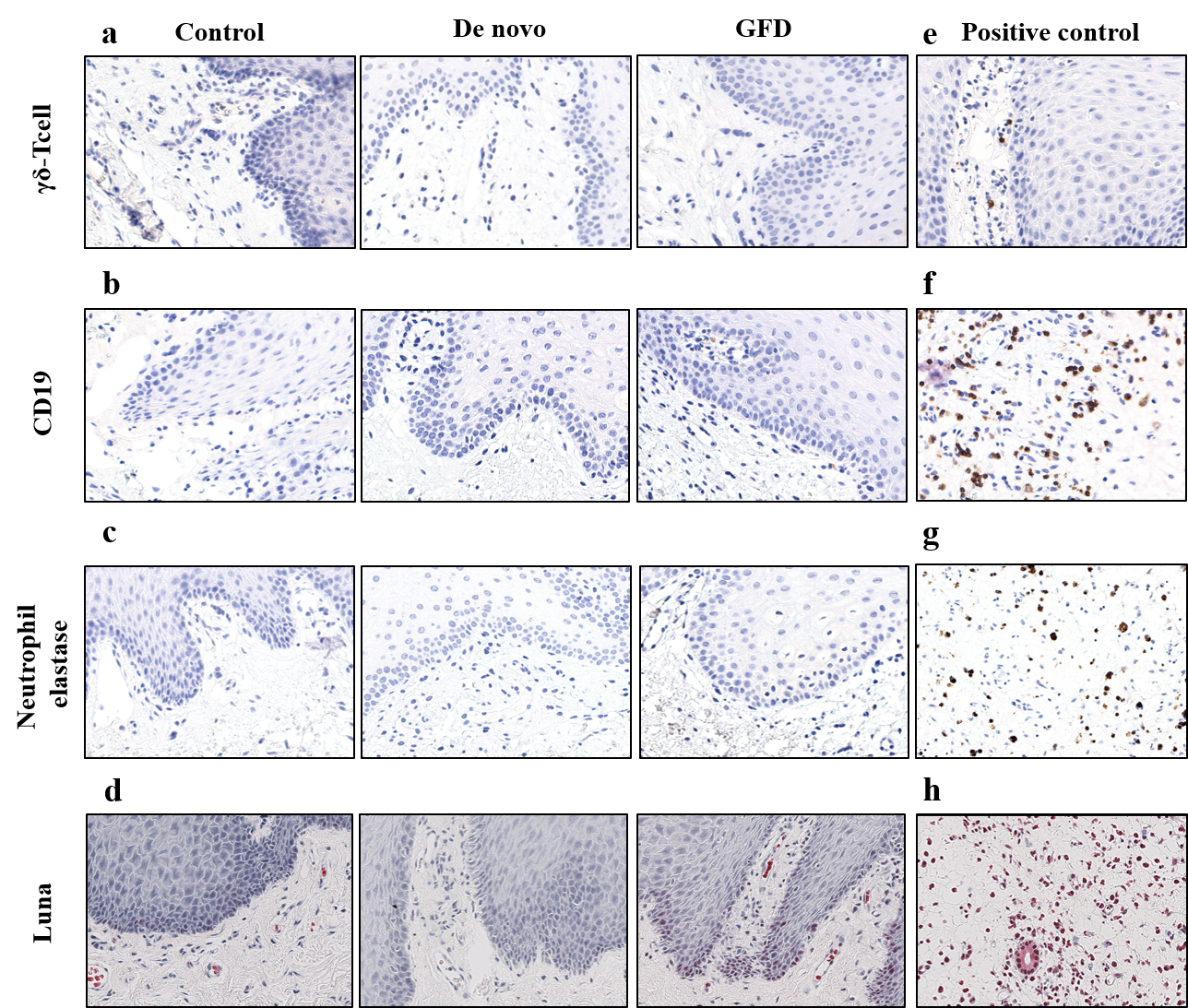


**Figure S2: Stains for γδ-T cell Receptor, CD19, Neutrophil elastase and Luna positive cell populations in the oral mucosa of control subjects and CD patients.**

Histochemical and immunohistochemical analyses of FFPE oral mucosal sections from non-celiac subjects (control) and CD patients *de novo* diagnosed and under GFD. Staining for γδ-T cell Receptor **(a)**, CD19 **(b)**, neutrophil elastase **(c)** and Luna (eosinophils) (d). No significant DAB signal is detected (a-c). Pink staining shows erythrocytes inside the blood vessels, no eosinophils are observed **(d)**. Oral mucosa from an atopic dermatitis patient was used as a positive control for γδ-T cells **(e)**. Nasal polyp served as positive control for CD-19 **(f)**, neutrophil elastase **(g)** and eosinophils **(h)**. All images were captured at 15X magnification.

**
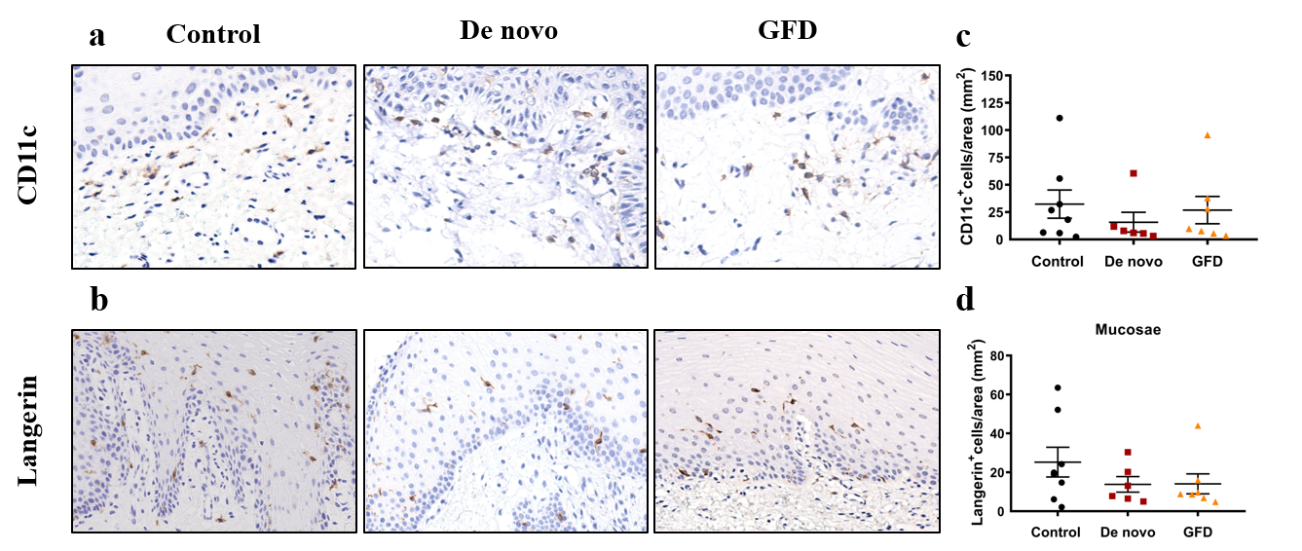
**

**Figure S3: Antigen Presenting Cell counts in the oral mucosa of control and celiac disease patients**

Representative images of **(a)** CD11c and **(b)** langerin immunohistochemistry of oral mucosal sections for different experimental groups. CD11c **(c)** and langerin **(d)** counts in overall mucosa for control subjects and CD patients *de novo* diagnosed and under GFD. Quantification is expressed as counts per area (mm2) of total mucosa. Images were captured at 20x magnification. Scatter plots show mean ± SEM.


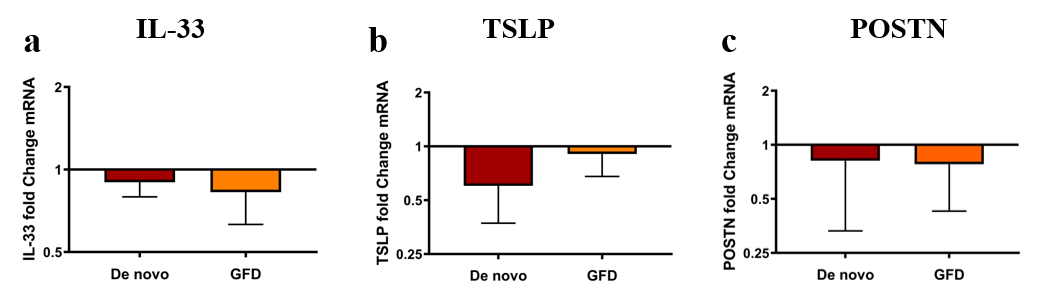


**Figure S4: mRNA fold change expression of epithelial alarmins in oral mucosa biopsies.**

mRNA fold change expression after RT-qPCR of RNA-later-embedded oral mucosa biopsies taken from the cheek lining of *de novo* diagnosed CD patients (De novo) and CD patients on GFD (GFD). IL-33 **(a),** TSLP **(b),** POSTN **(c)** observed fold-change using 2 -ΔΔCT method. Fold change is referred to non-celiac (control) samples. Bar plots show mean ± SD *P < 0.05, ** P<0.01.
